# Polymer-Based Membranes for Oily Wastewater Remediation

**DOI:** 10.3390/polym12010042

**Published:** 2019-12-26

**Authors:** Djamila Zioui, Hugo Salazar, Lamine Aoudjit, Pedro M. Martins, Senentxu Lanceros-Méndez

**Affiliations:** 1 Unité de Développement des équipements Solaires, UDES/Centre de Développement des Energies Renouvelables, CDER, Bou Ismail, W. Tipaza 42415, Algerie; ziouidjamila@yahoo.fr (D.Z.); lamineaoudjit@yahoo.fr (L.A.); 2 Centre/Departament of Physics, University of Minho, Campus de Gualtar, 4710-057 Braga, Portugal; hsalazar@fisica.uminho.pt; 3 Institute of Science and Innovation on Bio-Sustainability (IB-S), University of Minho, 4710-057 Braga, Portugal; 4 Laboratoire de Chimie du Gaz Naturel, Faculté de Chimie, BP 32, El Alia, U.S.T.H.B., Bab Ezzouar 16111, Algerie; 5 BCMaterials, Basque Centre for Materials, Applications and Nanostructures, UPV/EHU Science Park, 48940 Leioa, Spain; 6 IKERBASQUE, Basque Foundation for Science, 48013 Bilbao, Spain

**Keywords:** oil, nanocomposite membranes, PVDF-TrFE, photoreactor, water remediation

## Abstract

The compounds found in industrial wastewater typically show high toxicity, and in this way, they have become a primary environmental concern. Several techniques have been applied in industrial effluent remediation. In spite of the efforts, these techniques are yet to be ineffective to treat oily wastewater before it can be discharged safely to the environment. Membrane technology is an attractive approach to treat oily wastewater. This is dedicated to the immobilisation of TiO_2_ nanoparticles on poly(vinylidene fluoride–trifluoro ethylene) (PVDF-TrFE) porous matrix by solvent casting. Membranes with interconnected pores with an average diameter of 60 µm and a contact angle of 97°, decorated with TiO_2_ nanoparticles, are obtained. The degradation of oily wastewater demonstrated the high photocatalytic efficiency of the nanocomposite membranes: Under sunlight irradiation for seven hours, colourless water was obtained.

## 1. Introduction

The production of oil and gas by petroleum refineries yields a large amount of wastewater, containing mostly organic compounds (aliphatic and aromatic hydrocarbons), inorganic compounds (metals), and dissolved and suspended solids [1].

Nowadays, many techniques are being employed in oily wastewater treatment, such as ultrafiltration [2], ultrasonic separation [3], adsorption [4], and coagulation/flocculation [5], among others. However, these techniques present some drawbacks, such as the large space requirement, low cost and the generation of secondary pollutants. On the other hand, membrane processes are considered a suitable and low-cost alternative to the conventional techniques for treatment of oilfield wastewater, due to unique properties such as the simples process, low energy consumption and no phase transformation [6,7]. Recent studies have reported the use of polymeric membranes for oily wastewater treatment [8,9,10]. Zhang, B. et al. [11] reported the use of a polytetrafluoroethylene (PTFE) membrane for the study of the mechanisms of adsorption fouling of crude oil. Yagoub, H. et al. [12] fabricated a cellulose/chitin/chitosan/PET membrane to be applied in the treatment of oily wastewater. Yang, Y. et al. [13] developed a membrane using palygorskite with a potential application in oil/water separation. A work of Saththasivam, J. et al. [14] reported a new approach for a fast and efficient oil/water separation using a membrane based on ZnO microspheres and carbon nanotubes. However, membrane fouling has proven to be the major drawback of polymeric membranes use in oily wastewater treatment [11].

Furthermore, the hydrophobicity of polymeric membranes promotes their contamination in the filtration process, especially in oil/water separation, leading to the decrease of water flow and rejection efficiency [15]. Membranes based on PVDF have been investigated for the removal of different pollutants from water, such as copper ions [16], natural organic matter [17], proteins [18], organic compounds [19], volatile organic compounds [20] and desalination [21,22], among others. Of all the PVDF copolymers, poly(vinylidene fluoride-trifluoroethylene) (PVDF-TrFE) presents suitable physicochemical properties for photocatalytic applications, such as a high UV resistance, mechanical and chemical resistance and hydrolytic and thermal stability [23]. This polymer also allows for the production of membranes with controlled porosity and pore size. More recently, a titanium dioxide (TiO_2_)/PVDF-TrFE nanocomposite produced by the authors showed a remarkable solar photocatalytic activity in tartrazine degradation—degradation of 78% under five hours of sunlight irradiation [24].

As a catalyst, titanium dioxide (TiO_2_) is the most widely used photocatalyst for degradation of organic compounds due to its properties, such as non-toxicity, low cost and abundance, physical and chemical stability, superhydrophilicity, and superior photocatalytic activity under UV radiation (λ < 390 nm) [25]. The incorporation of TiO_2_ into polymeric membranes solves some drawbacks related to its use in suspension, namely the recuperation of the nanoparticles.

An efficient alternative to applying photocatalytic membranes are the photocatalytic membrane reactors (PMRs). PMRs have shown a great potential as a “zero” waste process for oily wastewater treatment since they can reduce the loss of catalytic nanoparticles, control the contact time between catalyst and pollutant, and guarantee a continuous process. In this context, PMRs can improve the process efficiency and stability, and reusability of the nanocomposite to reduce the operating cost [26].

Thus, the major focus of this work is to demonstrate TiO_2_ nanoparticles immobilised on a PVDF–TrFE porous membrane as being an efficient method for the degradation of oil in wastewater in a solar photoreactor, making use of the UV radiation (3–5%) present in the solar radiation.

## 2. Material and Methods

### 2.1. Materials

PVDF–TrFE (70:30) was obtained from Solvay (Paris, France). P25^®^-TiO_2_ nanoparticles were provided by EVONIK (Essen, Germany). Sulfuric acid (H_2_SO_4_) 99%, sodium hydroxide (NaOH) 97%, *N*,*N*-dimethylformamide (DMF) 99%, and methylene (MB) were all purchased from Merck (Darmstadt, Germany).

### 2.2. Membrane Preparation

The solvent casting technique was used to produce the PVDF-TrFE membranes with immobilised TiO_2_ nanoparticles [27,28]. Briefly, 0.86 g (8 wt.%) of P25-TiO_2_ nanoparticles generously provided by Evonik (Essen, Germany) were added to 90 mL of *N*,*N*-dimethylformamide (DMF) obtained from Merck (Darmstadt, Germany) and placed in an ultrasound bath for four hours to obtain a homogeneously dispersed solution. The amount of TiO_2_ used was previously chosen [28] to avoid nanoparticles detachment from the polymer matrix and to preserve its mechanical properties. Afterwards, 10 g of PVDF-TrFE 70:30 purchased from Arkema (Colombes, France) was added to the solution, achieving a concentration of 10 wt.% polymer to solvent, and the solution was magnetically stirred until complete polymer dissolution. Ultimately, the solution was placed in glass support, and the solvent evaporated at an ambient temperature. When the solvent completely evaporated, a membrane with the same dimensions of the photoreactor surface (38 cm length × 12 cm wide) was obtained.

### 2.3. Membrane Characterization

The crystal structure of the TiO_2_ nanoparticles was evaluated by X-ray diffraction (XRD) using a Bruker D8 Discover diffractometer with incident Cu Kα (40 kV and 30 mA). Fourier-transformed infrared spectroscopy (FTIR) was performed to determine the chemical stability of the nanocomposite using an FTIR Alpha instrument (Bruker Corporation, Billerica, MA, USA), over a range of 650–4000 cm^−1^ using 64 scans with a resolution of 4 cm^−1^. The scanning electron microscopy (SEM) was obtained with a Quanta 650 SEM (Thermo Fisher, Waltham, MA, USA) to access the morphology/microstructure of the membrane. Energy dispersive X-ray spectroscopy (EDX) was performed with an INCA 350 spectrometer (Oxford Instruments, Abingdon, UK), without gold coating.

The wettability of the samples was measured through contact angle in Physics SCA20 microscope (DataPhysics Instruments GmbH, Filderstat, Germany).

### 2.4. Wastewater Sample

The Hassi R’mel (HR) area is located in southern Algeria, 525 km south of Algiers, and 120 km south of Laghouat wilaya, about 70 km west of Beriane and 120 km south of Algiers, northwest of Ghardaia. The HR field is centered at 32° 55′41° North and 3° 16′16° East at an average elevation of approximately 750 m above sea level. The geometric shape, the nature of the effluent and the homogeneity of the reservoir at the HR field led to the selection of three production areas (North, Center, and South) and two compressor stations.

The withdrawal of samples was accomplished on 15 March 2017, at the inlet of the de-oiling unit at CPI S-102 (Corrugated Plate Interceptor), which is a separating basin furnished with a set of parallel plates that facilitate the sedimentation of the non-decanted solid particles. The samples were transported from Hassi R’mel to the Research and Development Center in coolers and stored for 24 h in a refrigerator at 4 °C before being used.

### 2.5. Oily Wastewater Parameters

Several parameters were measured before and after the treatment. The temperature (T) was measured using a thermometer ASTM 5C and the electrical conductivity (EC) was measured by a conductivity meter HACH HQ 40d. The suspended solids (SS) were determined by filtering 100 mL of water to be analyzed through a 47 μm pore filter; afterwards, the filter was dried at 105 °C in an oven for two hours. The amount of suspended solids was estimated by the difference in the weight of the filter before and after drying. The pH measurements were carried out using a pH-meter type (Inolab PH7310, Weilheim in Oberbayern, Germany). Chemical oxygen demand (COD) was based on the oxidation of organic materials by an excess of potassium dichromate (K2Cr2O7), in acidic medium and boiling, in the presence of silver sulfate and mercury sulfate. COD was measured by a spectrophotometer of the type (DR 1900 LANGE HACH, Duesseldorf, Germany). The percentage of degradation of COD was estimated using the following Equation (1).

(1) $$Degradation\left(\%\right)=\frac{C_{0}-C_{t}}{C_{0}}\times 100,$$

where *C*_0_ and *C_t_* are the initial concentration of oxygen and concentration at time *t*, respectively.

The quantification of the total hydrocarbons (HC) in water followed an analytical protocol implemented according to the water standard methods (solvent extraction, purification on florisil cartridge, and finally gas chromatography analysis coupled to a flame ionisation detector–Clarus 580 CPG/FID).

Total organic carbon (TOC) analysis was performed by TOC Analyzer Formacs HT. The determination of phosphate, nitrate, and nitrite was carried out using a DR1900 colourimetric spectrophotometer at a wavelength of 860 nm using the different LCK reagents.

### 2.6. Photocatalytic Degradation Measurement

**Figure 1:**
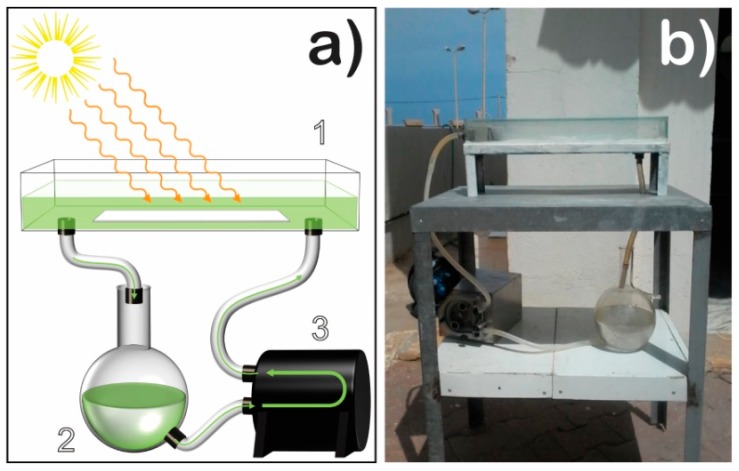
Representation of the solar photoreactor: (**a**) (1) photoreactor tank with the photocatalytic membrane at the bottom; (2) oily wastewater; (3) peristaltic pump; (**b**) a picture of the real photoreactor setup.

The photocatalytic degradation of oily wastewater was performed in a solar photoreactor located in the north of Algeria (latitude 36.39°; longitude 2.42° at sea level), using natural sunlight radiation. A Pyranometer CMP 11 (Kipp & Zonen, Delft, The Netherlands) with a spectral range between 285 and 2800 nm was used to measure the solar UV intensity. The reactor was developed at the Solar Equipment Development Unit (UDES) in Algeria. The capacity of the photoreactor is 1 L (38 cm length × 12 cm wide × 8.5 cm high). The photoreactor tank was fabricated from Pyrex glass, where the produced 8 wt.% TiO_2_/PVDF-TrFE nanocomposite membrane was placed at the bottom (Figure 1). The flow rate used to recirculate the oily water was 100.8 L h^−1^; in this way, it was possible to completely cover the nanocomposite membrane with the oily water. The photoreactor was wholly covered with glass to avoid evaporation during the photocatalytic experiments.

For the photocatalytic assays, one liter of oily wastewater solution was added to the photoreactor tank containing the membrane and exposed to solar illumination for seven hours, which is the period that assures a constant sunlight irradiance intensity during the experiment.

The degradation of oily wastewater was analyzed with a UV–visible spectrophotometer (Shimadzu-1800, Duisburg, Germany), and the peak at 262 nm was used to monitor oily wastewater absorbance over the irradiation time.

## 3. Results and Discussion

**Figure 2:**
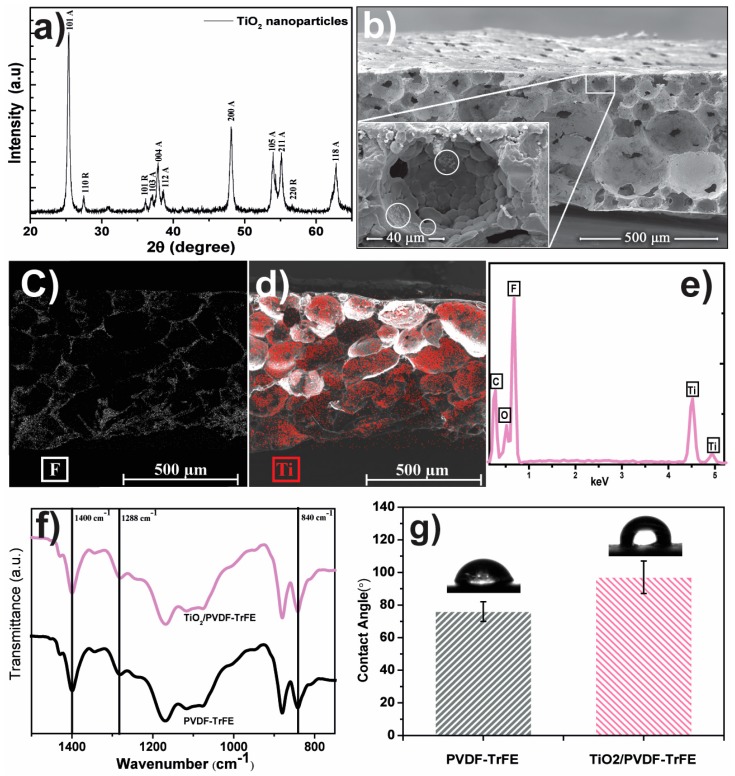
(**a**) XRD pattern of TiO_2_ nanoparticles and identification of the different crystal phases: Anatase (A), and rutile (R). (**b**) Cross-section SEM images of 8 wt.% TiO_2_/poly(vinylidene fluoride–trifluoro ethylene) (PVDF–TrFE) membranes with an inset showing a detail of an interconnected pores, with white circles for TiO_2_ nanoparticles and aggregates. SEM-EDX mapping image of the presence and distribution of F (fluorine) (**c**), and Ti (red) (**d**), in the PVDF-TrFE matrix and the EDX spectrum with the identification of the detected elements (**e**). FTIR spectra and contact angle of 8 wt.% TiO_2_/PVDF–TrFE nanocomposite membranes and pure polymer, (**f**,**g**) respectively.

The nanoparticles and membrane characterizations are summarized in Figure 2. The crystalline structure of TiO_2_ nanoparticles was assessed by X-ray diffraction (XRD) and compared with the pattern diffractograms (Figure 2a). The diffractogram indicates intense reflexes at 2θ ≈ 25.39° (101), 37.13° (103), 37.89° (004), 38.65° (112), 48.09° (200), 53.99° (105), 55.15° (211), and 62.81° (118), corresponding to the anatase phase (A), in agreement with the standard spectrum of anatase JCPDS 21-1272 [29,30,31] and JCPDS no.: 84-1286 [32]. In the same sample there are also diffraction values at 2θ ≈ 27.49° (110), 36.15° (101), and 56.65° (220) that are in agreement with the standard spectrum of the rutile phase (R) JCPDS 88-1175 [32].

SEM cross-section images, Figure 2b, shows the porous microstructure of the produced membrane with the characteristic interconnected (small pores inside the pores) spherical pores with an average diameter of ≈60 µm. Moreover, it is possible to identify TiO_2_ nanoparticles and agglomerates attached to the pore walls, signaled with circles in the inset of Figure 1b—SEM surface micrographs are available as supplementary material (Figure S1). The EDX mapping, Figure 2c,d, allows confirmation of the homogeneous distribution of titanium (Ti) and fluorine (F), ascribed to the presence of TiO_2_ nanoparticles over the porous PVDF-TrFE matrix, respectively. The EDX spectrum confirms the presence of all elements present on the nanocomposite, Figure 2e.

Infrared spectroscopy (FTIR) was used to determine the polymer phase and the possible chemical interaction between fillers and polymer matrix. In Figure 2c, the FTIR spectra of 8 wt.% TiO_2_/P(VDF–TrFE) nanocomposite show a stable polymer structure concerning the pure polymer. Bands at 840, 1288 and 1400 cm^−1^ show that the polymer crystallizes in the all-trans piezoelectric β-phase [27].

The polymer phase remains unchanged when TiO_2_ is present in the membranes, as already reported in Martins et al. [28]. In this way, filler content and type do not change the crystallization phase of the polymer, which crystallizes in the all-trans β-phase, and no chemical bonds are detected between polymer and fillers.

Contact angle measurements assessed the wettability of the membranes. Figure 2d illustrates the contact angle for the pristine and the 8 wt.% of TiO_2_/PVDF-TrFE samples, which present contact angles of 97° and 76°, respectively. These results show the decrease of the contact angle with the incorporation of TiO_2_ nanoparticles on the polymer matrix, which in turn, allows for a higher interaction between the nanocatalyst and the oily water.

### 3.1. Photocatalytic Degradation of Oily Wastewater

#### 3.1.1. Effect of Initial Chemical Oxygen Demand

Before the photocatalytic assays, controls were performed using oily wastewater without membrane (photolysis), and oily water with pristine PVDF-TrFE (adsorption), and in both cases the degradation was neglectable (≈1%)—see results as supplementary material Figure S2. The original oily water, with a COD value of 51,758 mg/L, was diluted with distilled water to obtain approximately half of the initial COD value ≈ 25,879 mg/L. Afterwards, both samples were placed on the solar photoreactor to perform a degradation under the same conditions. The obtained results are shown in Table 1.

This experiment has indicated an increased removal efficiency after diluting the initial oily water sample, with removal rates (R%) of 49% and 99.6% for the COD values of 51,758 and 25,879 mg/L, respectively. Such enhanced efficiency is related to turbidity reduction after dilution, from 425 NTU to 205 NTU. Decreasing turbidity favors the penetration of the sunlight radiation, which leads to oil removal rate improvement.

**Figure 3:**
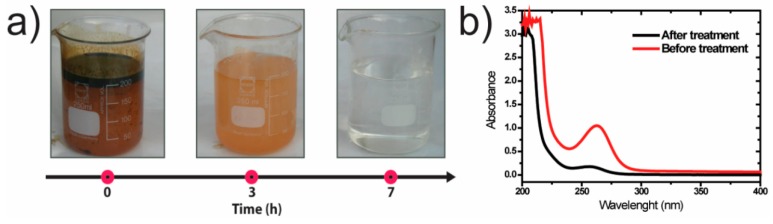
(**a**) The appearance of the oily wastewater along with the treatment with the 8 wt.% TiO_2_/P(VDF–TrFE) nanocomposite membrane in the solar photoreactor; (**b**) UV–vis absorption spectrum before and after the photocatalytic treatment of oily water after 7 h under sun irradiation/

The 8 wt.% TiO_2_/P(VDF–TrFE) membranes were used to assess the degradation of oily wastewater in a solar photoreactor. Figure 3a shows the shift of transparency of the oily water after 7 h under sunlight radiation.

The visual inspection indicates that the oily wastewater pollutants were degraded as colorless water was obtained after the seven hours. As confirmation, Figure 3b shows the absorption spectra of oily water samples before and after the photocatalytic treatment. The maximum absorption band is located at 262 nm. The decrease of the absorption peak at 262 nm confirms the photodegradation of oily water.

#### 3.1.2. Effect of Oily Water pH

In a different set of assays, the pH influence on the oily water degradation process was assessed; thus, three oily water solutions were prepared, and the initial pH was adjusted either with sulfuric acid (H_2_SO_4_) or sodium hydroxide (NaOH) solutions.

The results obtained indicate that the photocatalytic degradation is strongly related to the initial pH of the oily water solution. The highest removal efficiency, approximately 80%, was obtained at the initial pH of 5.5. Moreover, the results indicate that acid or basic solutions reduce the photocatalytic efficiency below 40% of COD removal.

#### 3.1.3. Wastewater Parameters

Additionally, other parameters that were measured for oily wastewater, before and after the treatment, are shown in Table 2.

**Figure 4:**
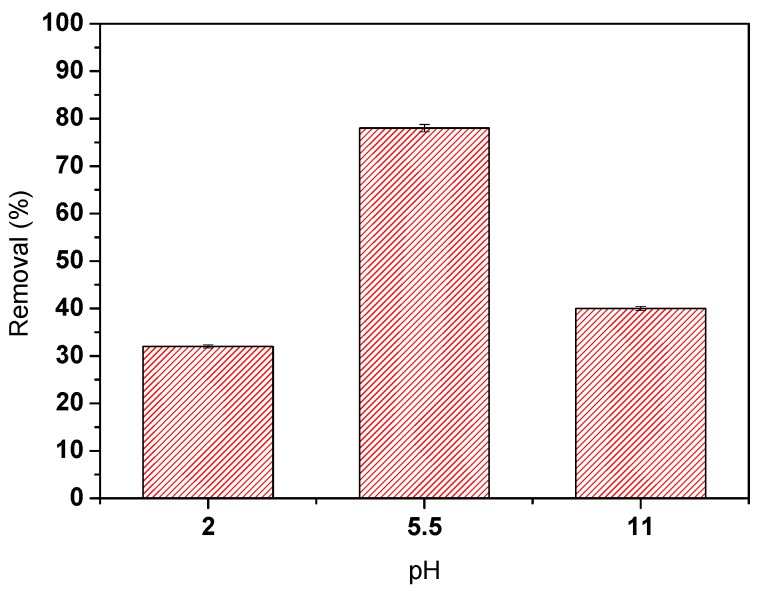
The influence of the initial pH on oily water photocatalytic degradation.

The results obtained after seven hours of sunlight irradiation of the 8 wt.% TiO_2_/PVDF-TrFE membrane in contact with the oily wastewater in the photoreactor show a COD removal around 99%. The suspended solids concentration was largely removed, and only 24% of the solids remained in the solution. There was also a significant decrease in the conductivity that is generally associated with nitrogen removal (in agreement with measured parameters) [33]. The total hydrocarbons (HC) were completely removed (>99%), which indicates the efficient removal of the oil in the wastewater. Furthermore, there was a reduction in all the assessed parameters, especially nitrate reduction, and all the final values were below the legal limits. It is also relevant to highlight that these measurements confirm that the highest COD removals are not obtained in extremely acidic or basic pH’s, as confirmed in the previous section (Figure 4).

The reusability of the TiO_2_/PVDF-TrFE was formerly proved in [24], which also contributes to making this material suitable for applications.

**Figure 5:**
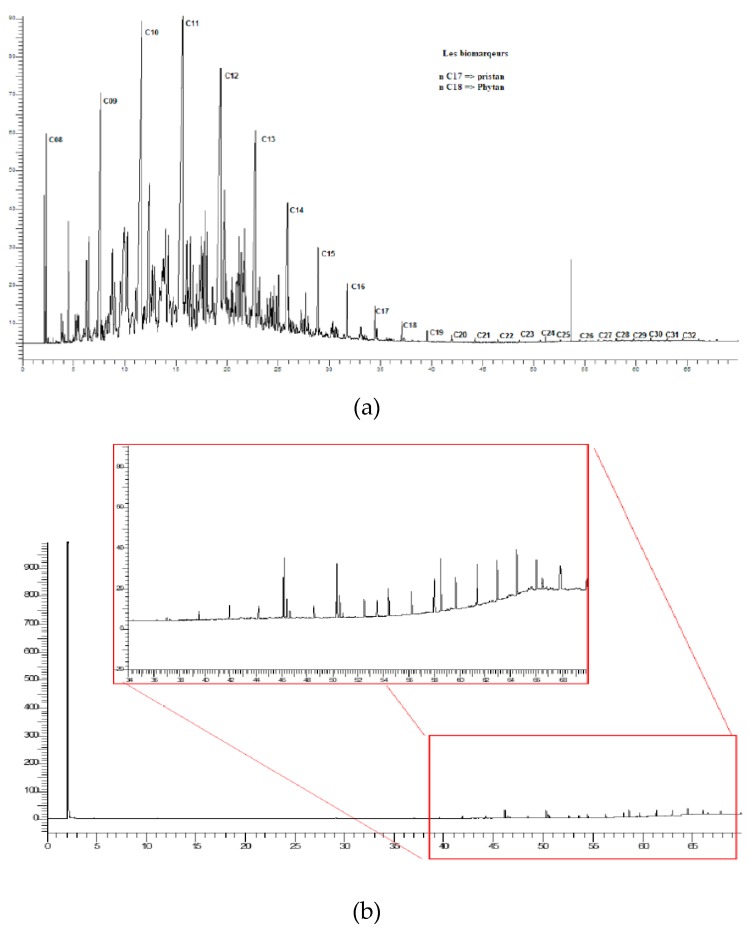
Chromatogram corresponding to the sample before (**a**) and after (**b**) the photocatalytic degradation in seven hours of sunlight irradiation.

The disappearance of peaks in the chromatogram obtained by gas chromatographic analysis (GC-FID) confirms the efficient removal of total hydrocarbons, Figure 5.

The majority of the organic compounds present in the sample before treatment (Figure 5a) belong to the alkanes family (C10-C30), which is in good agreement with similar works [6,34]. After the photocatalytic treatment, the presence of all these compounds was reduced, Figure 5b. The long-chain hydrocarbons were degraded by photocatalytic activity into smaller organic compounds.

The results show that by using the 8 wt.% TiO_2_/PVDF-TrFE nanocomposite membrane, it is possible to treat oily industrial wastewater, such as that from the central region of Hassi Rmel Algeria, using the sun as a source of energy.

## 4. Conclusions

This work reports the preparation of a TiO_2_/P(VDF–TrFE) nanocomposite by solvent casting and its photocatalytic application in the remediation of oily wastewater. The characterization of the samples shows a homogeneous distribution of the TiO_2_ nanoparticles over the pores with an interconnected structure. FTIR and contact angle results prove the chemical stability and the unchanged hydrophobic nature of the nanocomposite membrane. The photocatalytic assays allowed complete removal of the COD (99.6%), total hydrocarbons (99.6%), and phosphates (100%), as well as a significant reduction of the suspended solids (76%), turbidity (97%), TOC (98%), nitrates (69%), and chloride (26%) from oily wastewater. The pH range that allows better removal efficiencies ranges between 4 and 5.5. After seven hours of sunlight exposure, colourless oily wastewater was obtained. Thus, the fabricated membranes combine an inexpensive nanocatalyst and UV radiation source (available in sunlight radiation) to efficiently remediate oily water. Therefore, the TiO_2_/P(VDF–TrFE) nanocomposite membranes proved to possess a proper morphology and chemical stability envisaging oily wastewater remediation and related applications.

## Figures and Tables

**Table 1:** Chemical oxygen demand (COD) values and respective removal rate (%) for oily water after treatment in photoreactor with the 8% TiO_2_/P(VDF–TrFE) membrane after seven hours under sun irradiation.

COD (mg/L)	Removal Rate (%)
51,758	49
25,879	99.6

**Table 2:** Main parameters of the wastewater before and after the proposed treatment.

Parameter	Before Treatment	After Treatment	Removed (%)	Limit Values ^1^
pH	4.3	5.3	-	6.5–8.5
Temperature (°C)	21.1	19.1	9.47	30
Turbidity (NTU)	205	7	96.58	10
COD (mg L^−1^)	25879	105	99.59	120
Conductivity (ms cm^−1^)	236	167.4	29.06	2
Chloride (mg L^−1^)	1047.4	804.2	25.82	500
Suspended solids (mg L^−1^)	73.9	17.9	75.77	35
Total hydrocarbons (mg L^−1^)	48.69	0.1492	99.69	10
TOC (mg L^−1^)	872	19.81	97.72	20
Nitrate (mg L^−1^)	440	137	68.86	50
Nitrite (mg L^−1^)	0.99	0	100	1
Phosphate (mg L^−1^)	0.17	0	100	2

**^1^** Executive Decree No. 06-141 of 20 Rabie El Aouel 1427 corresponding to 19 April 2006, defining the limit values for discharges of industrial liquid effluents of Algeria.

## Supplementary Materials

The following are available online at https://www.mdpi.com/2073-4360/12/1/42/s1, Figure S1: Surface SEM image of a TiO2/PVDF-TrFE nanocomposite. Figure S2: Photocatalytic controls performed with just oily water, PVDF-TrFE, and the TiO2/PVDF-TrFE, under the same conditions.
