# Supplementary material for: Polymer-Based Membranes for Oily Wastewater Remediation
Source: Polymers (Basel). 2019 Dec 26;12(1):42. doi: 10.3390/polym12010042 (PMC7023582; doi:10.3390/polym12010042)
Supplement: Supplementary file 1 [file polymers-12-00042-s001.pdf]

Supplememntary material

Polymer-based membranes for oily wastewater remediation

D. Zioui[1†], H. Salazar[2,3†], L. Aoudjit[1,4], P. M. Martins[2,3*], and S. Lanceros-Méndez[2, 5,6*]

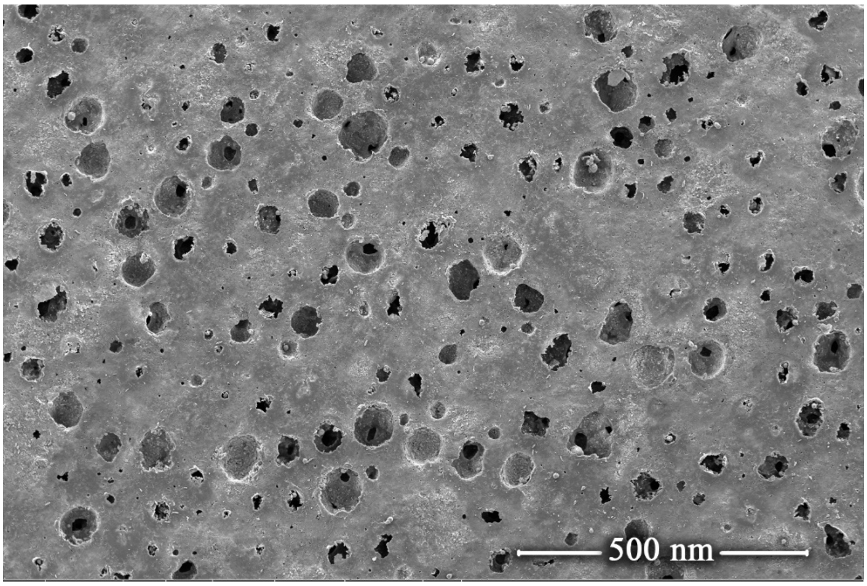

Figure S1. Surface SEM image of a TiO_2/PVDF-TrFE nanocomposite.

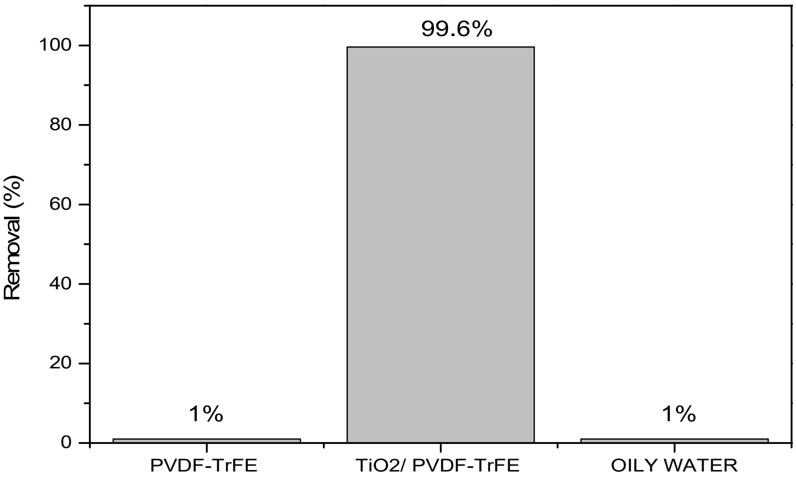

Figure S2. Photocatalytic controls performed with just oily water, PVDF-TrFE, and the TiO_2/PVDF-TrFE, under the same conditions.
